# The Effect of Oxygen on Phase Equilibria in the Ti-V System: Impacts on the AM Processing of Ti Alloys

**DOI:** 10.1007/s11837-018-3008-8

**Published:** 2018-07-11

**Authors:** Greta Lindwall, Peisheng Wang, Ursula R. Kattner, Carelyn E. Campbell

**Affiliations:** 1 Materials Science and Engineering Division, National Institute of Standards and Technology, 100 Bureau Dr., Stop 8555, Gaithersburg, MD 20899-8555 USA; 2 Department of Materials Science and Engineering, KTH Royal Institute of Technology, Brinellv. 23, 10044 Stockholm, Sweden; 3 Center for Hierarchical Materials Design (CHiMaD), Northwestern University, 2205 Tech Drive, Evanston, IL 60208 USA

## Abstract

Oxygen is always a constituent in “real” titanium alloys including titanium alloy powders used for powder-based additive manufacturing (AM). In addition, oxygen uptake during powder handling and printing is hard to control and, hence, it is important to understand and predict how oxygen is affecting the microstructure. Therefore, oxygen is included in the evaluation of the thermodynamic properties of the titanium-vanadium system employing the CALculation of PHAse Diagrams method and a complete model of the O-Ti-V system is presented. The *β*-transus temperature is calculated to increase with increasing oxygen content whereas the extension of the *α*-Ti phase field into the binary is calculated to decrease, which explains the low vanadium solubilities measured in some experimental works. In addition, the critical temperature of the metastable miscibility gap of the *β*-phase is calculated to increase to above room temperature when oxygen is added. The effects of oxygen additions on phase fractions, martensite and *ω* formation temperatures are discussed, along with the impacts these changes may have on AM of titanium alloys.

## Introduction

Titanium (Ti) has a large solubility of oxygen in both the *α* (hcp, hexagonal close-packed) and *β* (bcc, body-centered cubic) phases. A mole fraction of more than 0.30 oxygen can be dissolved in *α*-Ti before an oxide is formed, and, with increasing oxygen content, both the *α*- and the *β*-transus temperatures increase. Due to the large oxygen solubility, it is hence safe to assume that all titanium alloys manufactured from raw materials of nominal purity contain an non-negligible amount of oxygen. Titanium alloy powder used for powder-based additive manufacturing (AM) is not an exception. Furthermore, additional oxygen uptake during powder handling, printing and powder reuse is hard to control and, hence, it is important to understand and be able to computationally predict how oxygen is affecting the microstructure.

Numerous AM reports and roadmaps, see, e.g.,^1^–^4^ have pointed out the need for new or modified materials specially designed to better accommodate the AM processes compared to conventional grades. To speed up this process, and to meet today’s high demands for short time-to-market times, ICME (integrated computational materials engineering) is needed. To enable the ambitious ICME objectives, multicomponent computational thermodynamic data in terms of CALculation of PHAse Diagrams (CALPHAD) databases is instrumental. The CALPHAD method is a semi-empirical approach originally developed for the calculation of phase diagrams.^5^ The method is not limited to the thermodynamic properties and, nowadays, it is applied to describe several phase-based properties for materials such as atomic mobilities, molar volumes and elastic properties.^6^,^7^ The strength of the CALPHAD method is the possibility to extrapolate the properties of subsystems into multicomponent space and, hence, its usefulness for multicomponent computational thermodynamics. The current work focuses on the thermodynamics of the Ti-V system and the construction of a CALPHAD description to be used as a subsystem in a multicomponent titanium system. The aim is for this description to be applicable for real titanium alloys and their development. For this reason, oxygen is added to the Ti-V description. Emphasis is on titanium-rich systems and the simultaneous effect of vanadium and oxygen additions on the thermodynamics.

In the following sections, relevant literature information is reviewed, CALPHAD models are described, and the results are presented. Finally, oxygen effects on quantities important for the development and processing of titanium alloys such as transus-temperatures and phase fractions, are discussed with reference to powder-bed AM technologies.

## Thermodynamic Information in the Literature

### Ti-V

**Fig. 1:**
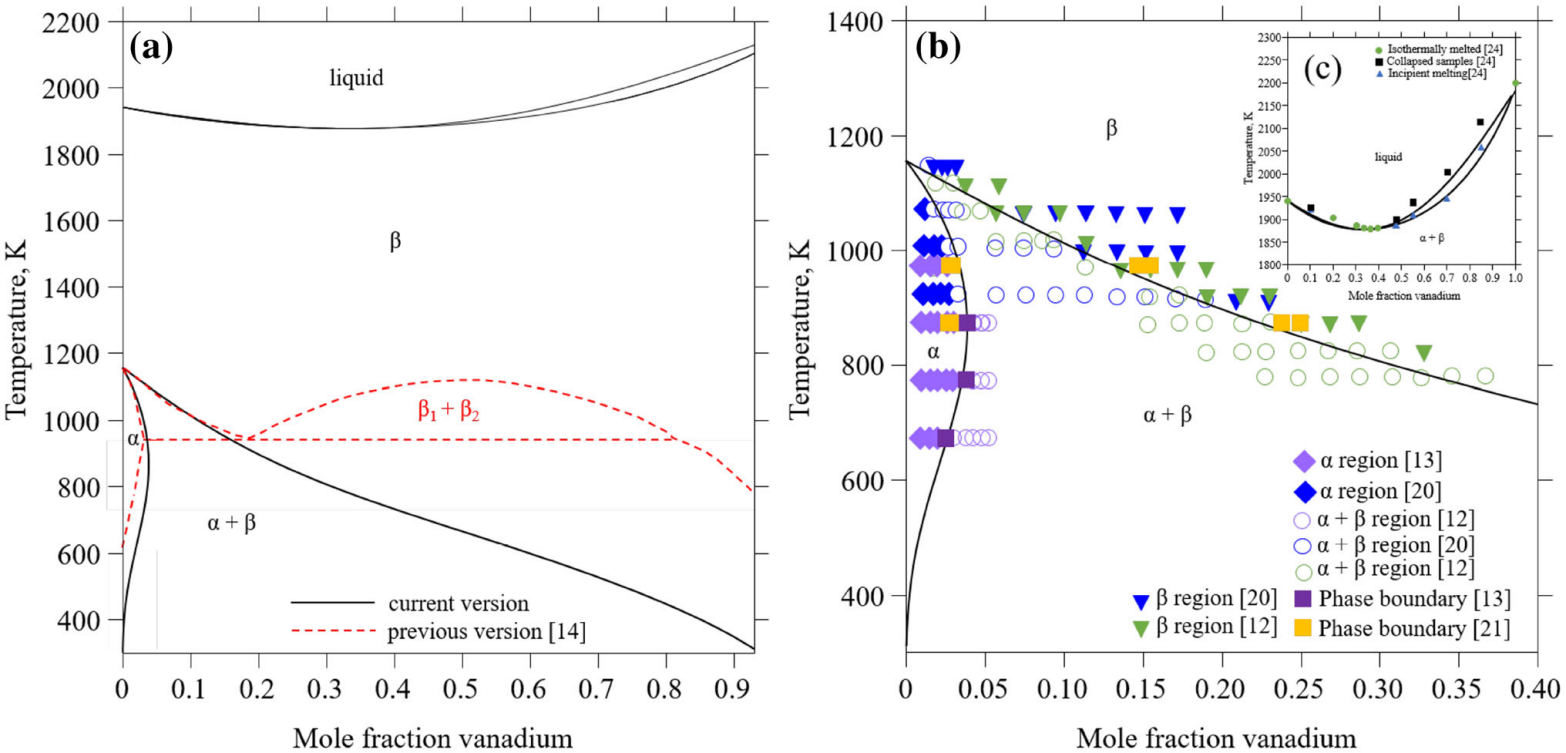
(a) The Ti-V phase diagram as reported in the literature: current version (solid lines) without and previous version (dashed lines) with a monotectoid β $\to$ *β*_1_ + *β*_2_ reaction. (b, c) Calculated Ti-V phase diagram in comparison to experimental data:^12^,^13^,^20^,^21^,^24^ (b) low temperature region and (c) high temperature region

The thermodynamics of the Ti-V system has been investigated multiple times and several CALPHAD descriptions are available.^8^–^10^ The reported phase diagrams show inconsistency and can be divided into two groups: one in which the miscibility gap in the *β*-(Ti,V) phase is metastable (Fig. 1, solid lines) and one where the miscibility gap is stable (Fig. 1, dashed lines). The phase diagram review by Murray 1981^11^ suggested that the *β*-transus decreases continuously with increasing vanadium content, based on experimental data by Ermanis et al.^12^ and Molokanov et al.,^13^ i.e., without a monotectoid reaction (*β* $\to$ *β*_1_ + *β*_2_). Murray concluded that oxygen contamination or insufficiently rapid cooling increased the *β*-transus temperature and, hence, relied on the data by Ermanis et al.^12^ in the evaluation. Later, Murray updated the Ti-V review^14^ and relied instead solely on the study by Nakano et al.^15^ and suggested a phase diagram that was characterized by a miscibility gap in the *β*-(Ti,V) phase with a critical temperature of 1123 K and the monotectoid temperature of 948 K. Nakano et al.^15^ used electrical resistivity measurements and phase analysis by x-ray diffraction analysis of samples with more than 0.10 mass fraction vanadium. Both methods showed evidence of a monotectoid reaction, *β* $\to$ *β*_1_ + *β*_2_, above 948 K. These results, however, are currently questioned due to a study by Fuming and Flower^16^ that pointed out that the Nakano et al.^15^ study did not report on the amount of impurities in their materials, in particular oxygen. Fuming and Flower showed in a new study using high-purity samples that there is no evidence of a monotectoid reaction (*β* $\to$ *β*_1_ + *β*_2_) but instead a stable *α* + *β* phase field with decreasing *β*-transus with increasing vanadium content, consistent with the diagram suggested by Murray.^11^ Fuming and Flower^16^ also studied Ti-V samples with various impurity levels of oxygen and concluded that increasing oxygen content widens the *β*_1_ + *β*_2_ miscibility gap and the stable *α* + *β* phase field. Consequently, for oxygen alone to be responsible for a monotectoid form of diagram, it must affect both the *α* and the *β* phases: i.e., oxygen increases the interaction parameter in the *β* phase^17^ and opens the miscibility gap while at the same time it decreases the free energy of the *α* phase.^18^ Nowadays, the simple Ti-V phase diagram with only three equilibrium phases (liquid, *α* and *β*) and no monotectic reaction is the widely accepted diagram. However, the inconsistencies in the reference literature has led to confusion, and, frequently, outdated Ti-V phase diagrams are being cited, e.g. Ref. 19.

The phase diagram debate described above mainly concerned higher vanadium compositions with a mole fraction ≥ 0.20. It is also important to note that the phase boundary data points on the titanium-rich side are scattered,^12^,^13^,^20^,^21^ as shown in Fig. 1b. This variation is believed to be caused by different levels of impurities (particularly oxygen). Of the available studies, only that by Molokanov et al.^13^ measured the vanadium solubility in *α*-Ti using ultra-pure titanium samples. Their results showed a maximum vanadium solubility with a mole fraction of ≈ 0.037 at 773 K and 873 K. The other works showing smaller vanadium solubility^12^,^20^,^21^ used material of less purity or unspecified purity.

In addition to experimental information for the solid phases in the Ti-V system, a couple of theoretical studies using ab initio methods^22^,^23^ are available. Uesugi et al.^22^ calculated the solution enthalpies using density functional theory (DFT) for both pure titanium (*α* and *β*) and pure vanadium (*α* and *β*), as well as for dilute solutions (*α*/*β* Ti_35_V_1_ and Ti_1_V_35_). Chinnappan et al.^23^ calculated the sub-solidus equilibrium phase diagram for Ti-V through cluster expansion, lattice dynamics and Monte Carlo methods combined with DFT and found agreement with CALPHAD assessed experimental phase boundaries without specifying the literature source further.

Experimental data involving the liquid are limited to solidus measurements by Adenstedt et al.^20^ using optical pyrometry, and to measurement of the titanium and vanadium activity in the liquid.^24^ To avoid reaction with the crucible, Mills and Kinoshita^24^ used a technique that involved electromagnetic levitation in an inert atmosphere. In both works, nominally pure titanium and vanadium were used and, therefore, the assertion that the influence of impurities is negligible cannot be assured.

Although the Ti-V equilibrium phase diagram is simple, with only three phases, phase transformations within the system can be complex and involve metastable phases. The *β* phase can transform to *α*′ martensite during quenching when richer in titanium or to *α*″ martensite when richer in vanadium.^25^,^26^ At higher vanadium contents, the *β* phase can also be retained as a metastable phase.^26^ Furthermore, during the *β* decomposition, the metastable phase *ω* can form as a transition phase.^26^ The formation of *ω* in quenched-in samples have been studied many times: e.g. using transition electron microscopy/scanning transition electron microscopy (TEM/STEM)^27^–^32^ and x-ray and neutron diffraction.^33^,^34^ The characteristics of the *ω* phase differ depending on the vanadium content in the alloy. The *ω* that forms at lower vanadium contents, i.e., less than a mole fraction of about 0.16, has a distorted bcc structure which has a hexagonal^34^ symmetry, whereas the one forming at higher vanadium contents shows less ω-specific reflections in the diffraction spectra.^32^,^35^ This can be explained by a partial collapse of the *β* structure instead of the complete collapse to the hexagonal structure. Moreover, Ghosh et al.^32^ concluded that the interface between *ω* and *β* is coherent, and that the transformation occurs under two different conditions: firstly, athermally during quenching, and secondly, isothermally during heat treatment, resulting in an elliptical shape of the *ω* precipitates.

The martensite start temperatures (*M*_s_) and the *ω* start temperatures (*ω*_s_) for the Ti-V system have been measured by several groups,^36^–^43^ and martensite and *ω* formation has been studied theoretically.^44^–^47^ The experimental values for the *M*_s_ temperature and its composition dependency are relatively consistent, whereas the measurements for *ω*_s_ scatter noticeably. One reason for the scatter is discussed by Paton and Williams,^42^ who identified the presence of impurities as one of the sources along with the wide range of techniques being used. Yan and Olson^46^ have developed a modeling approach and constructed a thermodynamic description of the *ω* phase in the Ti-V system. For this, they performed differential scanning calorimetry and dilatometry experiments as well as first-principles calculations for substitutional ordered alloys, using the virtual crystal approximation (VCA) and the local self-consistent Green’s function method based on exact muffin-tin orbitals (EMTO).^46^ From this, they could predict at which vanadium composition *T*_0_ (temperature of equal Gibbs energies at equal compositions of two phases) is located at 0 K for both the *β* $\to$ *α* and the *β* $\to$ *ω* transitions. Also, Leibovitch and Rabinkin^31^ modeled the ω phase based on room-temperature observations.

### Ti-O

Several thermodynamic descriptions and phase diagram evaluations^48^–^53^ are available for the Ti-O system, and a detailed review of all existing thermochemical and phase equilibria information will not be repeated here. However, a thorough discussion about the available information on the titanium-rich side of the system, particularly on the *α*- and *β*-transus temperatures and their oxygen dependencies, is motivated due to its importance for the processing of titanium alloys.

**Fig. 2:**
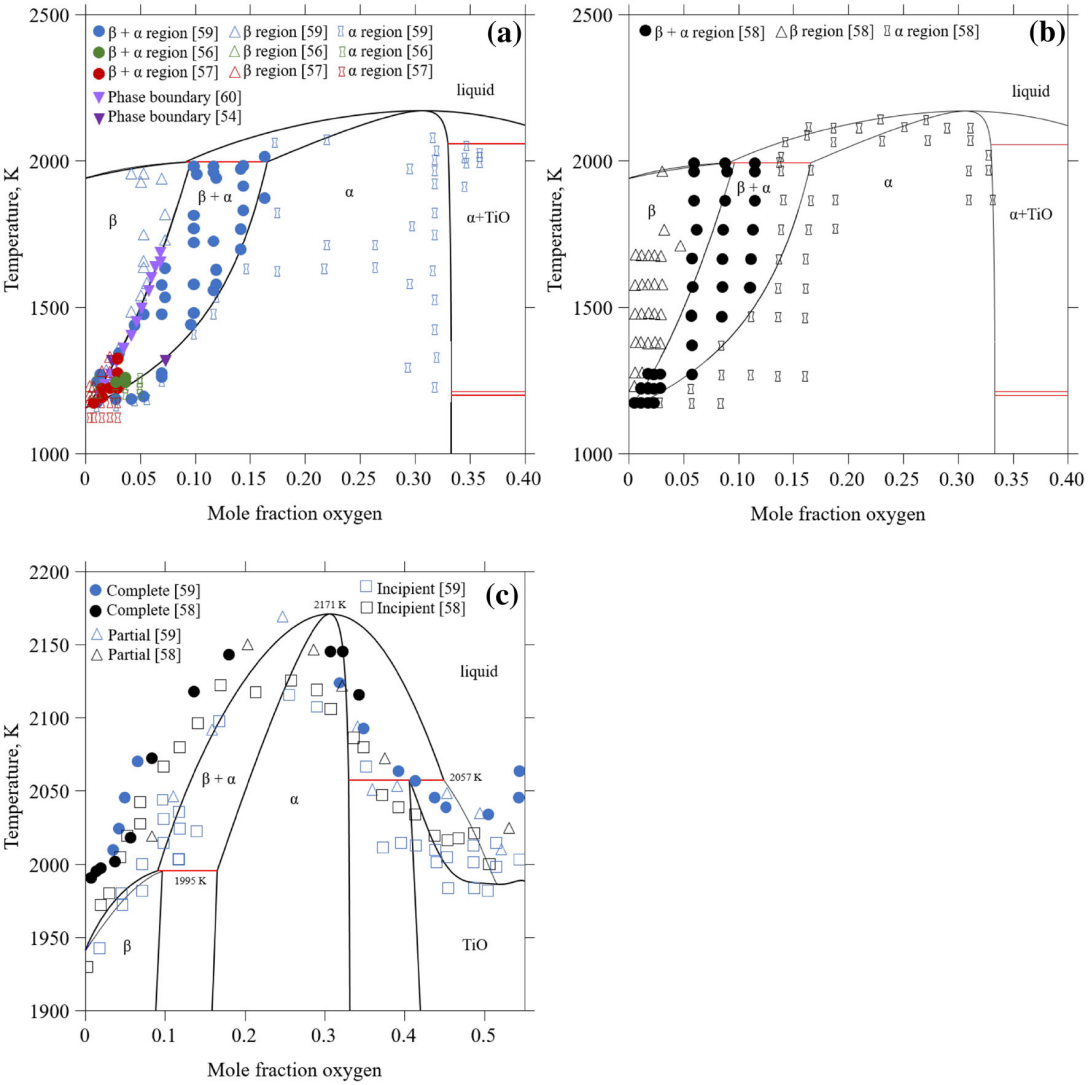
Calculated Ti-O phase diagram in the titanium-rich region in comparison to experimental data for the solid phase regions (a) and (b), and for the phase regions at higher temperatures including the liquid phase (c)^54^,^56^–^60^

In Fig. 2a and b, the different experimental datasets for the phase boundaries in the *α*/*β* region of the phase diagram are shown.^54^–^61^ There are eight works published of which the newest is from 1978 by Tetot et al.^54^ This phase boundary determination is limited to 1323 K and is a by-product of a microcalorimetric measurement of the partial molar enthalpy of oxygen in titanium. Tetot et al.^54^ concluded that their thermochemical measurements differed substantially from earlier studies, which they explained by the higher precision of their measurements. Kubaschewski and Dench^55^ and Mah et al.^61^ used thermochemical measurements to determine the phase boundaries, but at 1473 K, and located them at much smaller oxygen contents compared to Tetot et al.^54^ In addition, the phase boundaries have been determined by metallography,^57^–^59^ thermoelectric power measurements^56^ and diffusion couple experiments.^60^ In the evaluation of the Ti-O system by Murray,^52^ the data by Jenkins and Worner^56^ was preferred for the low-temperature part of the *α*-transus. For higher temperatures, above 1573 K, the two sets of data by Bumps et al.^58^ and Schofield and Bacon^59^ scatter and, according to Murray, there is no clear basis for preferring the data of either. The phase boundary determinations for the *β*-transus by Schofield and Bacon (Fig. 2a) and Bumps et al. (Fig. 2b) are also in disagreement with each other, and the measurements by Schofield and Bacon show higher oxygen solubility in *β*-Ti, particularly at higher temperatures. However, the results by Schofield and Bacon agree with the results of the diffusion studies by Wasilewski and Kehl,^60^ which also show higher oxygen *β* solubility then the Bumps et al. data. The dataset by Schofield and Bacon^48^ is also generally in better agreement with the data by Jenkins and Worner,^56^ Jaffee et al.^57^ and Tetot et al.^54^ than Bumps et al.^58^ Furthermore, Schofield and Bacon paid careful attention to impurity levels of the raw materials, used longer treatment times and better temperature control compared to the study by Bumps et al.,^58^ which is expected to increase the accuracy. It should be noted, however, that large error bars are expected for both datasets and that the error increases with increasing temperature.

*α*-Ti melts congruently at temperatures around 2173 K at approximately 0.24 mol fraction oxygen, according to optical pyrometric melting data.^58^,^59^ Optical pyrometric measurements were also used by Bumps et al. and Schofield and Bacon to determine the peritectic reaction liquid + *α*-Ti $\leftrightarrow$ *β*-Ti. According to Bumps et al., it occurs at 2013 K ± 25 K, and according to Schofield and Bacon at 1993 K ± 25 K. Here, the temperatures measured by Schofield and Bacon are believed to be more accurate as their measurement of the melting temperature of pure titanium (1933 K) is in better agreement with the accepted Ti melting temperature of (1941 K) than the one measured by Bumps et al. (1998 K). Both works used optical pyrometric measurements to determine the solidus and liquidus positions. Such measurements are difficult and the accuracy is expected to be low, in particular for the liquidus positions since the liquid reacted with the molybdenum crucible that they used. Schofield and Bacon used this method to determine the liquidus for oxygen contents above 0.15 mass fraction. In addition, for oxygen contents below 0.25 mass fraction, they determined the liquidus by observing the temperature at which liquid appeared in a deep axial hole in the alloy compact, and below 0.03 mass fraction oxygen, they used hardness measurements carried out on the core of the compacts, which had been liquid. This is a method that depends on the relationship between hardness and oxygen content of as-melted alloys.

The solidus was measured by Bumps et al^58^ also using the optical pyrometric technique and by metallographic examination of annealed samples. The error bar for the solidus was estimated by the authors to be ± 25 K which should be considered as approximate since their melting temperature of pure titanium is about 50 K off the actual melting temperature of titanium. In conclusion, little is known about the phase equilibria at high temperatures for the Ti-O system, and the available liquidus and solidus data has low accuracy and should only be considered as approximate in the modeling.

The thermochemical properties of *α*-Ti are relatively well determined: Hepworth and Schuhmann^62^ measured the titanium activity, Mah et al.^61^ and Ariya et al.^63^ reported on the enthalpy of formation, and Komarek and Silver^64^ measured the O_2_ partial pressure for *α*-Ti solid solutions. In the case of *β*-Ti solid solutions, the O_2_ partial pressure has been measured by a number of groups.^55^,^65^–^68^ All these datasets are in relative good agreement^69^ and, as will be shown in next section, they are also coherent with the *α*- and *β*-transus temperatures suggested by Schofield and Bacon, Jenkins and Worner, and Wasilevski and Kehl.^56^,^59^,^60^

Thermodynamic properties of the Magnéli oxides (Ti_*n*_O_2*n*−1_) are all well determined and are accurately assessed in the literature.^48^–^51^,^53^ In the current work, as well as by Hampl and Schmid-Fetzer,^48^ Cao et al.,^50^ Cancarevic et al.,^49^ Fisher^53^ and Waldner and Eriksson,^51^ these oxides are described as line compounds. The halite phase (Ti_1_O_1_, NaCl structure), on the other hand, shows a large solubility on both sides of the stoichiometric composition which should be accounted for. Experimental evidence exists that vacancies occupy both titanium and oxygen lattice positions,^70^ although little is known about the different Ti-cations and their site lattice occupancy.

### V-O

In the current work, the CALPHAD thermodynamic description of the V-O system published by Yang et al.^71^ is adopted without modification. Details on experimental and calculated input information are thoroughly reported on by Yang et al. and will not be repeated here.

### Ti-V-O

Recently, a CALPHAD description of the Ti-V-O system was published by Yang et al.^72^ This report focuses mainly on the properties of the Ti-V oxides in the system and includes a highly satisfying summary of the available thermodynamic information. The current work aims to complement the description by Yang et al. with a critical evaluation of the titanium-rich part of the Ti-V system and the effect of oxygen additions. We will therefore mainly concentrate here on the literature data available for the titanium-rich corner. As mentioned in the previous section, the thermodynamic properties of the Ti-V system have shown to be strongly dependent on the impurity levels in the studied samples. Due to the characteristics of the Ti-O phase diagram and the difficulties of completely eliminating the presence of oxygen in practice, oxygen can be regarded as a major impurity in all titanium alloys. Despite its unavoidable presence, few studies on titanium alloys include quantitative information about the oxygen content. In addition, other, often not quantified, impurities such as nitrogen and iron are expected to strongly influence the phase boundaries in titanium systems. This complicates a quantitative assessment of the Ti-V-O system in the titanium corner.

From the experimental studies reviewed in previous sections, it can be concluded that the presence of oxygen decreases the vanadium solubility in *α*-Ti in addition to decreasing the *β*-transus temperature.^17^ Furthermore, as mentioned previously, oxygen is believed to be one of the reasons for the disagreeing Ti-V phase diagrams in the literature.^16^

Several isothermal sections and isopleths in the Ti-V-O system have been published.^73^,^74^ Most of these studies focus on higher oxygen levels and the thermodynamic properties of the oxides. For oxygen contents, low enough for the alloy to be in the *α*/*α *− *β*/*β* phase regions, only two isothermal sections by Komjathy^75^ at 1073 K and 1473 K are noted. In that study, the focus was on vanadium-rich alloys and the phase boundary of single-phase *β*-V was determined metallographically and by using x-ray diffraction. It can be concluded that the oxygen solubility in this phase increases with increasing temperature. Few experimental details were reported by Komjathy, and their results for the binary V-O alloys are not in agreement with the V-O phase diagram.^71^ Hence, the accuracy of this information is hard to evaluate.

The transformation of *ω* also shows a dependency on oxygen content. Early studies by Ageyev et al.^76^ showed that the *ω* particle size was strongly affected by the amount of oxygen present, where the ω precipitates were much smaller in contaminated samples compared to the purer samples. In addition, Paton and Williams^42^ concluded that the ω transformation temperature was lowered by the oxygen presence. Oxygen may also play a role in the *ω* $\to$ *α* transformation. The work by Li et al.^77^ indicates that oxygen-rich regions present at the *ω*/*β* interface serve as sites for *α* formation, as they found oxygen-rich regions in the *α* phase in close proximity to the *ω* phase.

## Thermodynamic Modeling

The sublattice models for the oxides are adopted from Yang et al.^71^ (V-O), from Hampl and Schmid-Fetzer^48^ (Ti-O), and Yang et al.^72^ (Ti-V-O). The model parameters^48^,^71^,^72^ are kept intact, except for some revisions of the halite phase necessary to accommodate the new *α* and *β* descriptions.

The metastable *ω* phase expected to form as a transition phase in the titanium-richer systems is modeled as a hexagonal phase using the same sublattice model as for the *α* phase. The *ω* end-member compounds for the pure elements $^{0} G_{\text{Ti:Va}}$ and $^{0} G_{\text{V:Va}}$ are adopted from the description developed by Yan and Olson.^46^ The oxygen-containing ω end-members are constructed by assuming the energy difference between the oxygen *ω* end-member and the pure element is the same as this energy difference for the *α* phase; i.e.

1 $$ G_{{i:{\text{O}}}}^{\omega } = G_{{i:{\text{Va}}}}^{\omega } + G_{{i:{\text{O}}}}^{\alpha } - G_{{i:{\text{Va}}}}^{\alpha } $$

where *i* is titanium or vanadium. The zero-order interaction parameter for the *ω* phase assessed by Yan and Olson^46^ is adjusted by accounting for the T_0_ temperature for the *β* → *ω* transformation at 0 K predicted from first-principles.^46^

The model parameters for the Ti-V-O system resulting from the current work are listed in Table I. For the assessment, the optimization module PARROT included in the Thermo-Calc software package is utilized. The descriptions by Hampl and Schmid-Fetzer^48^ and Yang et al.^71^,^72^ of the oxide phases that are kept unmodified are not included in the table.

**Table I:** Parameters for the thermodynamic description of the phases in the Ti-V-O system in units Joule per mole formula and Kelvin

$$ {\text{LIQUID: (Ti}}^{ + 2} , {\text{V}}^{ + 2} )_{\text{P}} \left( {{\text{O}}^{ - 2} , {\text{Va}}^{{ - {\text{Q}}}} , {\text{O,TiO}}_{ 2} , {\text{TiO}}_{ 3 / 2} , {\text{VO}}_{ 2} , {\text{VO}}_{ 3 / 2} , {\text{VO}}_{ 5 / 2} } \right)_{Q} $$
$$ ^{0} G_{{{\text{Ti}}^{ + 2} :{\text{O}}^{ - 2} }} - H_{\text{Ti}}^{\text{SER}} - 2H_{\text{O}}^{\text{SER}} = 2 \cdot {\text{GTIOT}} + 155343.6 - 6.14 \cdot T $$
$$ ^{0} G_{{{\text{Ti}}^{ + 2} :{\text{Va}}^{ - Q} }} - H_{\text{Ti}}^{\text{SER}} = {\text{GLIQTI}}^{*} $$
$$ ^{0} G_{{{\text{V}}^{ + 2} :{\text{O}}^{ - 2} }} - H_{\text{V}}^{\text{SER}} - 2H_{\text{O}}^{\text{SER}} = 2 \cdot {\text{GL}}\_{\text{V}}1{\text{O}}1 $$
$$ ^{0} G_{{{\text{V}}^{ + 2} :{\text{Va}}^{ - Q} }} - H_{\text{Ti}}^{\text{SER}} = {\text{GLIQV}}^{*} $$
$$ ^{0} G_{{{\text{TiO}}_{2} }} - H_{\text{Ti}}^{\text{SER}} - 2 \cdot H_{\text{O}}^{\text{SER}} = {\text{GTIO2}} + 61022.4 - 28.2 \cdot T $$
$$ ^{0} G_{{{\text{TiO}}_{3/2} }} - H_{\text{Ti}}^{\text{SER}} - 1.5 \cdot H_{\text{O}}^{\text{SER}} = 0.5 \cdot {\text{GTI2O3}} + 57073.7 - 22.8 \cdot T $$
$$ ^{0} G_{{{\text{VO}}_{2} }} - H_{\text{V}}^{\text{SER}} - 2 \cdot H_{\text{O}}^{\text{SER}} = {\text{GL}}\_{\text{V}}1{\text{O}}2 $$
$$ ^{0} G_{{{\text{VO}}_{3/2} }} - H_{\text{V}}^{\text{SER}} - 1.5 \cdot H_{\text{O}}^{\text{SER}} = 0.5 \cdot {\text{GL}}\_{\text{V1O2}} + 3308.6 $$
$$ ^{0} G_{{{\text{VO}}_{5/2} }} - H_{\text{V}}^{\text{SER}} - 2.5 \cdot H_{\text{O}}^{\text{SER}} = 0.5 \cdot {\text{GL}}\_{\text{V2O5}} $$
$$ ^{0} L_{{{\text{Ti}}^{ + 2} :{\text{O}}^{ - 2} ,{\text{Va}}}} = 196881.943 - 121.808911 \cdot T $$
$$ ^{1} L_{{{\text{Ti}}^{ + 2} :{\text{O}}^{ - 2} ,{\text{Va}}}} = 216547.034 - 100.230047 \cdot T $$
$$ ^{0} L_{{{\text{V}}^{ + 2} :{\text{O}}^{ - 2} ,{\text{Va}}}} = - 98900 $$
$$ ^{1} L_{{{\text{V}}^{ + 2} :{\text{O}}^{ - 2} ,{\text{Va}}}} = 51034 $$
$$ ^{0} L_{{{\text{V}}^{ + 2} :{\text{O}}^{ - 2} ,{\text{VO}}_{3/2} }} = 1449.9 - 50.71 \cdot T $$
$$ ^{0} L_{{{\text{Ti}}^{ + 2} ,{\text{V}}^{ + 2} :{\text{Va}}}} = - 487 $$
$$ ^{1} L_{{{\text{Ti}}^{ + 2} ,{\text{V}}^{ + 2} :{\text{Va}}}} = 2730.8 $$
$$ ^{0} L_{{{\text{O}}^{ - 2} ,{\text{VO}}_{2} }} = 50000 $$
$$ ^{0} L_{{{\text{O}}^{ - 2} ,{\text{VO}}_{3/2} }} = 50000 $$
$$ ^{0} L_{{{\text{O}}^{ - 2} ,{\text{VO}}_{5/2} }} = 50000 $$
$$ ^{0} L_{{{\text{TiO}}_{2} ,{\text{TiO}}_{3/2} }} = - 19200.8 $$
$$ ^{1} L_{{{\text{TiO}}_{2} ,{\text{TiO}}_{3/2} }} = 100402.4 - 48.6 \cdot T $$
$$ ^{0} L_{{{\text{TiO}}_{2} ,{\text{VO}}_{3/2} }} = - 33000 $$
$$ ^{1} L_{{{\text{VO}}_{2} ,{\text{TiO}}_{3/2} }} = - 46000 $$
$$ {\text{BCC\_A2:(Ti,V)}}_{1} ({\text{O,Va}})_{3} $$
$$ ^{0} G_{\text{Ti:O}} - H_{\text{Ti}}^{\text{SER}} - 3H_{\text{O}}^{\text{SER}} = {\text{GHSERTI}}^{*} + 3 \cdot {\text{GHSEROO}} - 9 6 4 1 0 0+ 2 5 7. 4 4\cdot T $$
$$ ^{0} G_{\text{Ti:Va}} - H_{\text{Ti}}^{\text{SER}} = {\text{GBCCTI}}^{*} $$
$$ ^{0}G_{\text{V:O}} - H_{\text{V}}^{\text{SER}} - 3H_{\text{O}}^{\text{SER}} = {\text{GHSERV}}^{*} + 3 \cdot {\text{GHSEROO}} - 5 1 5 3 7 8+ 1 8 6. 9 3\cdot T $$
$$ ^{0}G_{{\text{V}}:{\text{Va}}}-H_{{\text{V}}}^{{\text{SER}}} = {\text{GHSERVV}}^{*} $$
$$ ^{0} L_{\text{Ti:O,Va}} = - 6 2 5 7 4 4. 9 3 8 $$
$$ ^{0} L_{\text{V:O,Va}} = - 5 1 3 3 7 6 $$
$$ ^{1} L_{\text{V:O,Va}} = 2 7 4 6 7 7 $$
$$ ^{0} L_{\text{Ti,V:Va}} = \;^{0} L_{\text{Ti,V:O}} = 3 4 2 7. 6 9 8 { + 1} . 2 4 8\cdot T $$
$$ ^{1} L_{\text{Ti,V:Va}} = \;^{1} L_{\text{Ti,V:O}} = 1 4 0 7. 8 6 1 $$
$$ {\text{HCP\_A3:(Ti,V)}}_{1} ({\text{O,Va}})_{0.5} $$
$$ ^{0} G_{\text{Ti:O}} - H_{\text{Ti}}^{\text{SER}} - 0.5H_{\text{O}}^{\text{SER}} = {\text{GHSERTI}}^{*} + 0.5 \cdot {\text{GHSEROO}} - 2 7 4 5 0 0+ 3 9. 5 1\cdot T $$
$$ ^{0} G_{\text{Ti:Va}} - H_{\text{Ti}}^{\text{SER}} = {\text{GHSERTI}}^{*} $$
$$ ^{0} G_{\text{V:O}} - H_{\text{V}}^{\text{SER}} - 0.5H_{\text{O}}^{\text{SER}} = {\text{GHCPVV}}^{*} + 0.5 \cdot {\text{GV1O1}} - 0.5 \cdot \,{\text{GFCCVV}}^{*} + 1 0 0 0 0 0\equiv {\text{GHVO}} $$
$$ ^{0} G_{\text{V:Va}} - H_{\text{V}}^{\text{SER}} = {\text{GHCPVV}}^{*} $$
$$ ^{0} L_{\text{Ti,V:Va}} = \;^{0} L_{\text{Ti,V:O}} = 1 8 5 0 4.1 - 9. 5 0 4\cdot T $$
$$ \omega : ( {\text{Ti,V)}}_{1} ({\text{O,Va}})_{0.5} $$
$$ ^{0} G_{\text{Ti:O}} - H_{\text{Ti}}^{\text{SER}} - 0.5H_{\text{O}}^{\text{SER}} = {\text{GTIOMG}} + {\text{GHTIO}} - {\text{GHSERTI}}^{*} $$
$$ ^{0} G_{\text{Ti:Va}} - H_{\text{Ti}}^{\text{SER}} = {\text{TIOMG}} $$
$$ ^{0} G_{{\text{V}}:{\text{O}}}-H_{{\text{V}}}^{{\text{SER}}}-0.5H_{{\text{O}}}^{{\text{SER}}} = {\text{GHVO}} + {\text{GHSERVV}}^{*} + 8 9 6 5-{\text{GHCPVV}}^{*} $$
$$ ^{0}G_{\text{V:Va}} - H_{\text{V}}^{\text{SER}} = {\text{GHSERVV}}^{*} + 8965 $$
$$ ^{0} L_{\text{Ti,V:Va}} = \;^{0} L_{\text{Ti,V:O}} = 4000 $$
$$ {\text{HALITE: }}\big( {{\text{Ti,Ti}}^{ + 2} , {\text{Ti}}^{ + 3} , {\text{V,V}}^{ + 2} , {\text{V}}^{ + 3} , {\text{Va}}} \big)_{ 1} \big( {{\text{O}}^{ - 2} , {\text{Va}}} \big)_{ 1} $$
$$ ^{0} G_{{{\text{Ti:O}}^{ - 2} }} - H_{\text{Ti}}^{\text{SER}} - H_{\text{O}}^{\text{SER}} = 3000 - 0.05 \cdot T + 0.5 \cdot {\text{GHSERTI}}^{*} + 0. 5\cdot {\text{GTIOX}} $$
$$ ^{0} G_{{{\text{Ti}}^{ + 2} :{\text{O}}^{ - 2} }} - H_{\text{Ti}}^{\text{SER}} - H_{\text{O}}^{\text{SER}} = {\text{GTIOX}} $$
$$ ^{0} G_{{{\text{Ti}}^{ + 3} :{\text{O}}^{ - 2} }} - H_{\text{Ti}}^{\text{SER}} - H_{\text{O}}^{\text{SER}} = 0.5 \cdot {\text{GTI}}2{\text{O}}3 + 158885.45 + 1 5. 7 1 3 4\cdot T $$
$$ ^{0} G_{{{\text{V:O}}^{ - 2} }} - H_{\text{V}}^{\text{SER}} - H_{\text{O}}^{\text{SER}} = {\text{GHCCVV}}^{*} - 30 \cdot T $$
$$ ^{0} G_{{{\text{V}}^{ + 2} :O^{ - 2} }} - H_{\text{V}}^{\text{SER}} - H_{\text{O}}^{\text{SER}} = {\text{GV1O1}} $$
$$ ^{0} G_{{{\text{V}}^{ + 3} :{\text{O}}^{ - 2} }} - H_{\text{V}}^{\text{SER}} - H_{\text{O}}^{\text{SER}} = 0.5 \cdot G{\text{V2O3 + 10956}} $$
$$ ^{0} G_{{{\text{Va::O}}^{ - 2} }} - H_{\text{O}}^{\text{SER}} = 0 $$
$$ ^{0} G_{\text{Ti:Va}} - H_{\text{Ti}}^{\text{SER}} = {\text{GHSERTI}}^{*} + 6000 - 0.1 \cdot T $$
$$ ^{0} G_{{{\text{Ti}}^{ + 2} : {\text{V}}a}} - H_{\text{Ti}}^{\text{SER}} = 0.5 \cdot {\text{GHSERTI}}^{*} + 3000 - 0.05 \cdot T $$
$$ ^{0} G_{{{\text{Ti}}^{ + 3} : {\text{Va}}}} - H_{\text{Ti}}^{\text{SER}} = {\text{GHSERTI}}^{*} + 6000 - 0.1 \cdot T $$
$$ ^{0} G_{\text{V:Va}} - H_{\text{V}}^{\text{SER}} = {\text{GFCCVV}}^{*} $$
$$ ^{0} G_{{{\text{V}}^{ + 2} :{\text{Va}}}} - H_{\text{V}}^{\text{SER}} = {\text{GV1O1 + 30}} \cdot T $$
$$ ^{0} G_{{{\text{V}}^{ + 3} :{\text{Va}}}} - H_{\text{V}}^{\text{SER}} = 0.5 \cdot {\text{GV}}2{\text{O}}3 + 10956 + 3 0\cdot T $$
$$ ^{0} G_{\text{Va::Va}} = 30 \cdot T $$
$$ ^{0} L_{{{\text{Ti,Ti}}^{ + 2} :{\text{O}}^{ - 2} }} = - 66167.5869 + 15.167789 \cdot T $$
$$ ^{0} L_{{{\text{Ti}}^{ + 2} ,{\text{Va:O}}^{ - 2} }} = - 339888 + 7.3928 \cdot T $$
$$ ^{0} L_{{{\text{Ti}}^{ + 3} ,{\text{Va:O}}^{ - 2} }} = - 4 7 1 4 6 2- 9. 8 9 4 4\cdot T $$
$$ ^{0} L_{{{\text{Ti}}^{ + 3} :{\text{O}}^{ - 2} ,{\text{Va}}}} = - 4 3 6 0 7 6+ 4 2. 0 6 7 4\cdot T $$
$$ ^{0} L_{{{\text{V}}^{ + 2} ,{\text{V}}^{ + 3} :{\text{O}}^{ - 2} }} = - 7 9 5 8. 4 $$
$$ ^{0} L_{{{\text{V}}^{ + 3} :{\text{O}}^{ - 2} ,{\text{Va}}}} = + 3 8 0 0 2 $$
$$ ^{0} L_{{{\text{V}},{\text{V}}^{ + 2} :{\text{O}}^{ - 2} , {\text{Va}}}} = + 3 7 2 0 5. 2 2 9 $$
$$ ^{0} L_{{{\text{Ti,V}}^{ + 2} :{\text{O}}^{ - 2} }} = + 2 0 0 0 0 0 $$
$$ ^{0} L_{{{\text{Ti,V}}^{ + 3} :{\text{O}}^{ - 2} }} = + 2 0 0 0 0 0 $$
$$ ^{0} L_{{{\text{Ti}}^{ + 2} ,{\text{V}}^{ + 2} :{\text{O}}^{ - 2} }} = + 5 0 0 0 0 $$
$$ ^{0} L_{{{\text{Ti}}^{ + 2} , {\text{V}}^{ + 3} :{\text{O}}^{ - 2} }} = + 5 0 0 0 0 $$
$$ ^{0} L_{\text{Ti,Va:Va}} = + 2 0\cdot T $$
$$ ^{0} L_{{{\text{Ti,V}}^{ + 2} :{\text{Va}}}} = + 2 0 0 0 0 0 $$
$$ ^{0} L_{{{\text{Ti,V}}^{ + 3} :{\text{Va}}}} = + 2 0 0 0 0 0 $$

*The end-member compound energies are from SGTE and can be found in the Pure elements database (PURE4) distributed by Thermo-Calc or in the paper by Dinsdale.^84^

The functions for the oxides rutile, corundum, *α*-TiO, *β*-V_3_O, M_4_O_7_, M_6_O_11_, M_7_O_13_, M_8_O_15_, Ti_10_O_19_, Ti_20_O_39_, Ti_2_O_5_, Ti_3_O_2_, Ti_3_O_5_, Ti_5_O_9_, Ti_9_O_17_, V_2_O_5_, V_2_O, V_3_O_5_, V_3_O_7_, V_52_O_64_, V_5_O_9_, V_3_O_13_, and VO_2_ can be found in the publications by Yang et al.^71^,^72^ and Hampl and Schmid-Fetzer,^48^ respectively.

### The Ti-V System

The calculated Ti-V phase diagram using the CALPHAD description developed in this work is shown in Fig. 1. To assess the interaction parameters for *α* and *β*, the phase boundary data by Molokov et al.,^13^ the 0 K *T*_0_ composition by Yan and Olson^46^ and the formation energies of Uesugi et al.^22^ are accounted for, as well as the liquids/solidus information by Adenstedt et al.^20^ In particular, the phase boundary data by Molokov^13^ is prioritized due to the caution taken concerning the purity of the samples used for this study.

The calculated maximum solubility of vanadium in *α*-Ti is a mole fraction of 0.0385 and is located at 830 K. At 773 K and 873 K, the calculated vanadium solubility is 0.0369 mol fraction, which is in exact agreement with the measured values for high-purity samples^13^ (~ 0.037 mol fraction). The *β*-transus temperature decreases continuously with increasing vanadium amount, which is in acceptable agreement with the measurements^12^,^21^,^22^ shown in Fig. 1b.

**Fig. 3:**
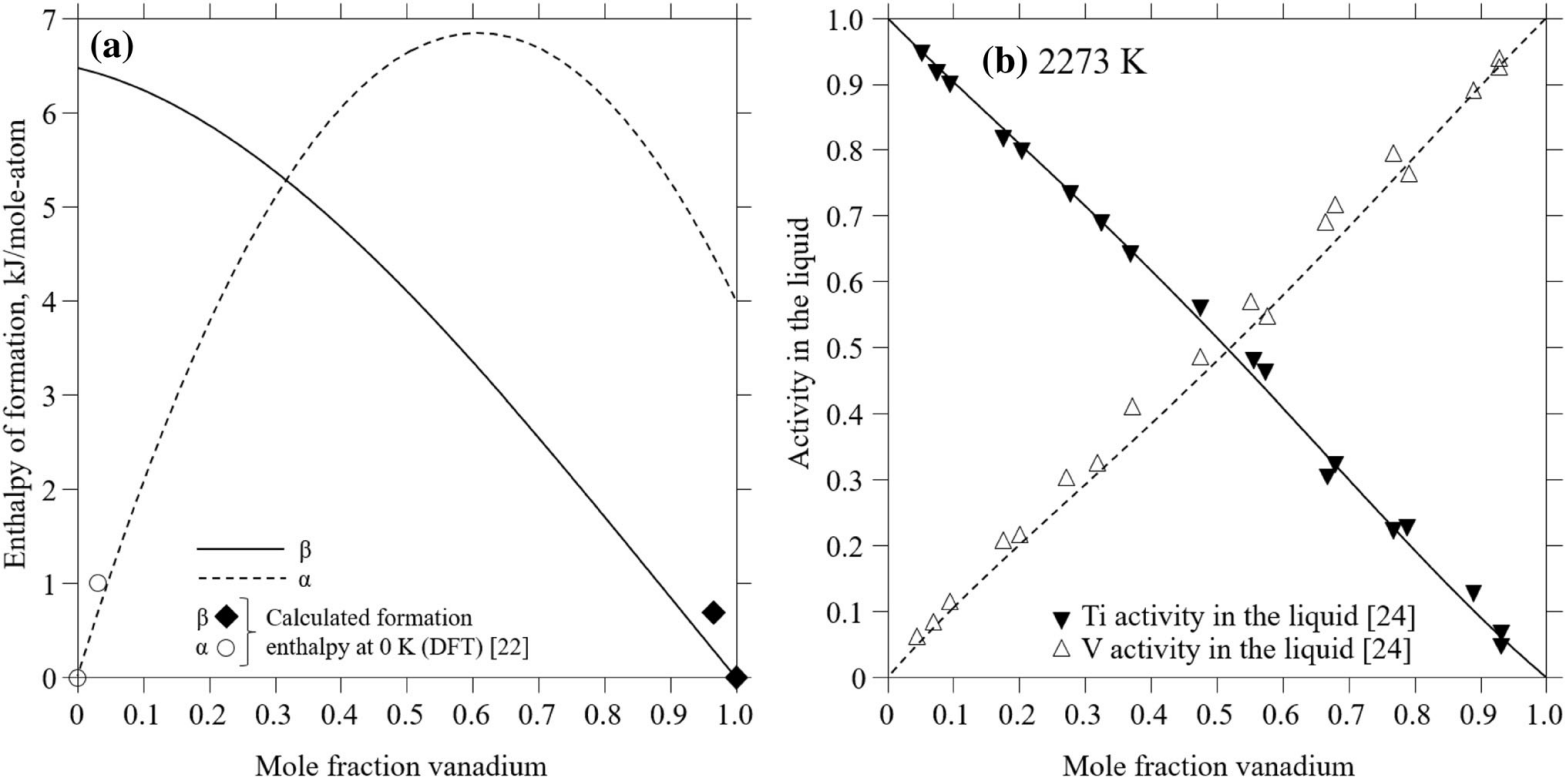
(a) Calculated enthalpy of formation as a function of vanadium concentration for the *α* and *β* phase at 298.15 K in comparison to DFT results (0 K) by Uesugi et al.^22^ (b) Calculated activities of vanadium and titanium in the liquid at 2273 K in comparison to experiments^24^

The calculated formation enthalpies at 298 K for the *α* and *β* phases are shown in Fig. 3a in comparison with DFT values at 0 K by Uesugi et al.^22^ for pure phases and dilute compositions (Ti_35_V_1_), and reasonable agreement is concluded. The agreement between the calculated titanium and vanadium activities in the liquid and the experimental results^24^ at 2273 K is shown in Fig. 3b.

**Fig. 4:**
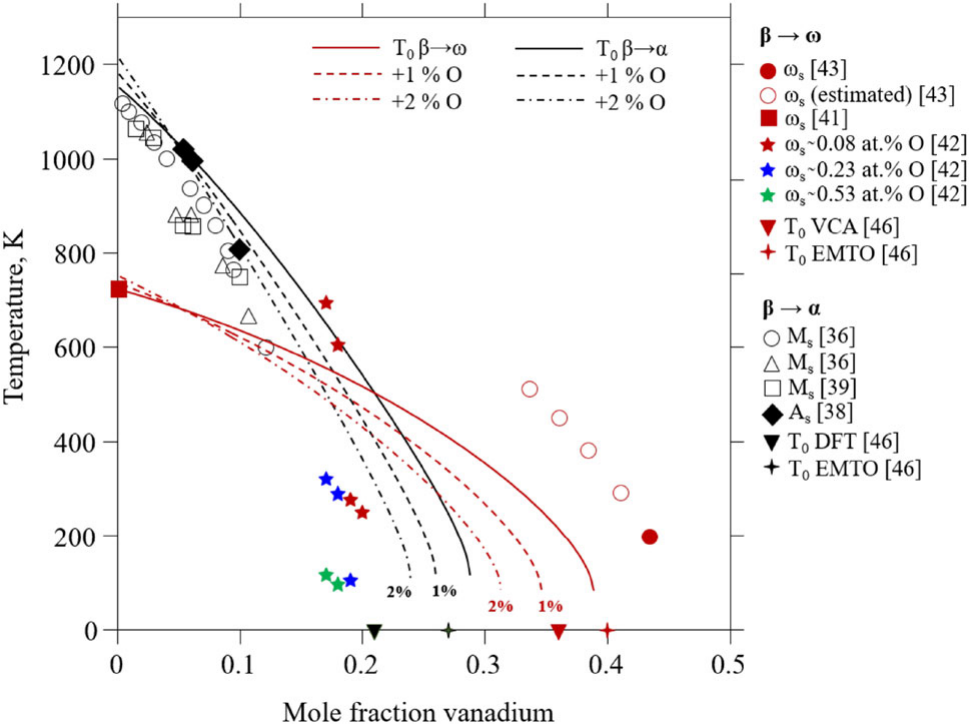
Calculated *T*_0_ temperatures for the *β*-*α* and *β*-*ω* transformations in comparison with experimental *M*_s_, *A*_s_, and *ω*_s_ temperatures.^36^–^39^,^41^–^43^ The oxygen amount is given in mole fraction. The 0 K *T*_0_ values are from first-principles calculations.^46^

In Fig. 4, the *T*_0_ temperatures calculated using the current description are shown in comparison to measured *M*_s_, the parent *β* start temperature (*A*_s_) and *ω*_s_ temperatures for the *β* $\to$ *α* and *β* $\to$ *ω* transitions. The vanadium compositions at the 0 K *T*_0_ temperature calculated from first-principles are also shown in Fig. 4 for the two transformations. Due to its definition, the *T*_0_ temperature must be above the measured *M*_s_ temperature but below the measured *A*_s_ temperatures. As can be seen in Fig. 4, this is achieved for the *β* $\to$ *α* transition using the developed description. The vanadium content at the 0 K *T*_0_ temperature is somewhat higher than suggested by both the VCA and EMTO calculations.^46^ This is a compromise to reproduce the measured *α*-*β* phase boundaries at higher temperatures. The different experimental datasets for the *ω*_s_ temperature show large scatter and no attempts are made to fit to any of them. The first-principles calculations for the 0 K *T*_0_ temperature by Yan and Olson,^46^ however, is used to revise the ω description for the Ti-V system. As expected, the *β *→ *ω T*_0_ curve crosses the *β *→ *α T*_0_ curve with increasing vanadium content. The large scatter is most likely due to impurities. This is further discussed in the section on the Ti-V-O system.

### The Ti-O System

The titanium-rich side of the Ti-O phase diagram is shown in Fig. 2a and b. In Fig. 2a, the calculations are shown in comparison with all the experimental *α*-*β* phase boundary data available except the data by Bumps et al.^58^ Good agreement is achieved for most cases: the calculated *α*-transus exactly reproduces the boundary measured by Wasilewski and Kehl,^49^ Tetot et al.^43^ and Schofield and Bacon,^54^,^59^,^60^ and is in close agreement with the measurements by Jaffee et al.^43^ and Jenkins and Worner.^56^ The calculated *α*-transus is also in exact agreement with the measurement by Tetot et al. and is in good agreement with Jaffee et al. and Jenkins and Worner. Both the calculated *α*- and *β*-transus temperatures are consistent with the work by Schofield and Bacon, especially at higher temperatures. The maximum oxygen solubility in the *β* phase with a calculated mole fraction of 0.096 occurs at 1995 K. This can be compared to the invariant reaction temperature for “liquid + *α* ↔ *β*″, 1993 K, measured by Schofield and Bacon.^59^ In Fig. 2b, the same calculation is instead compared with the data by Bumps et al. In the critical evaluation of the Ti–O system by Murray and Wriedt,^52^ it was stated that there is no obvious preference for the contradicting datasets by Schofield and Bacon and Bumps et al. However, in the current work, the same conclusion is drawn as by Cao et al.^50^ in their assessment of the Ti-C-O system, i.e., that the *α* and *β* descriptions that reproduce the experimental data for the thermochemical properties satisfactory are also consistent with all the measured phase boundaries except the ones by Bumps. Therefore, in this work, the *α* and *β* parameters by Cao et al.^50^ are adopted.

As discussed in the previous section, the behavior of the Ti-O system in the liquid regions is not well understood. The two experimental works available show considerable scatter and the error bars for both measurements are expected to be large. The measured melting temperature of pure Ti by Bumps et al.^58^ is off by about 50 K and, hence, caution should be taken to all their reported values, as this indicates that the titanium they used might have been considerably contaminated by impurities. For this reason, the current model is made to reproduce the congruent melting point of *α*, ~ 2170 K, as suggested by Schofield and Bacon.^59^ It is calculated to be located at 0.306 mol fraction oxygen which is higher than that suggested by Schofield and Bacon^59^ as well as by Bumps et al. Figure 2c shows that the measured liquids and solidus are poorly reproduced by the current model. Attempts to reproduce both the lower and higher temperature phase boundaries were made, but no satisfying compromise is possible. Due to the large uncertainty in the measurement in this high-temperature region of the diagram, this disagreement is accepted and good agreement with the phase boundaries for the solid *α* and *β* phases at lower temperatures together with the thermochemical information of the solid phases are instead prioritized.

### The Ti-V-O System

**Fig. 5:**
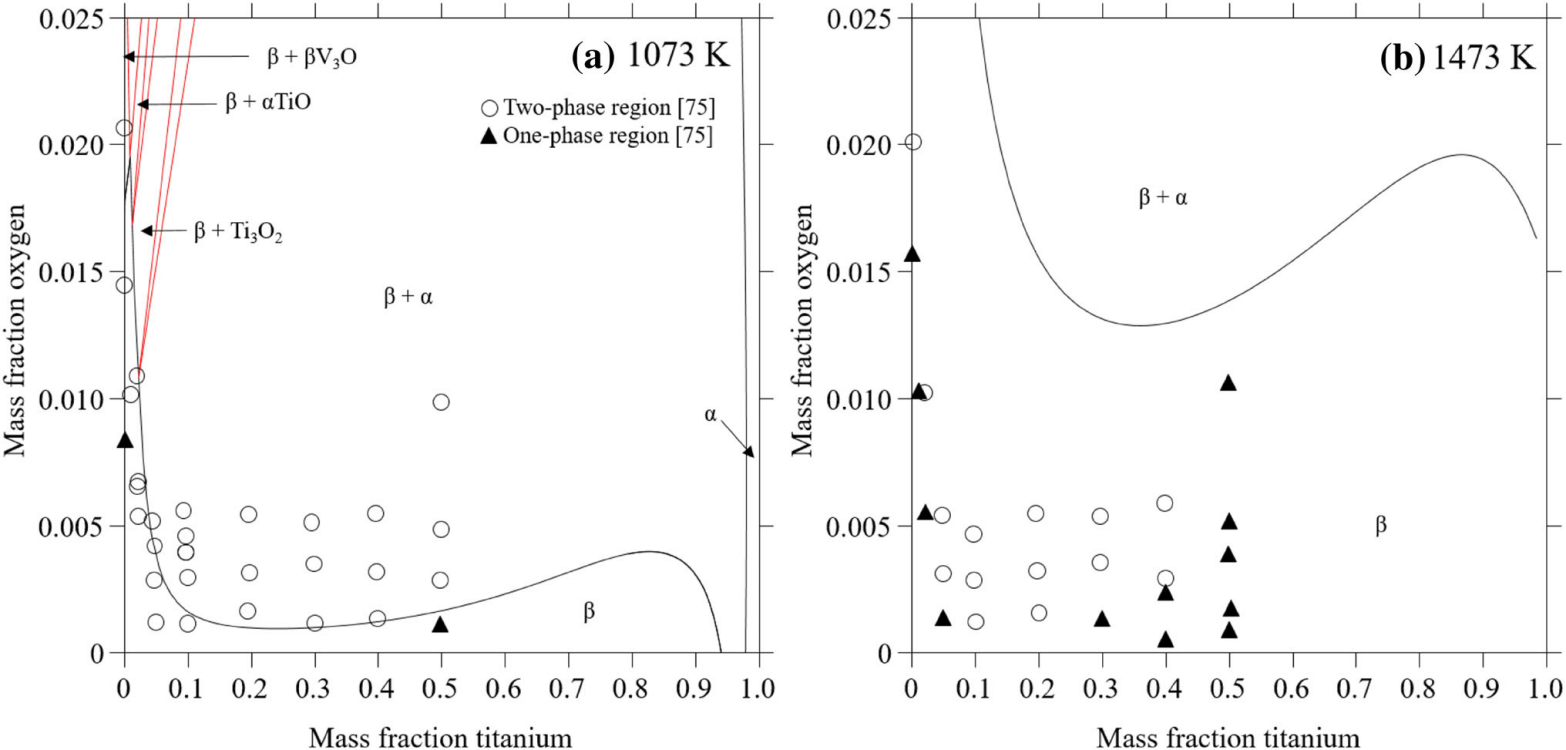
Calculated isothermal sections at (a) 1073 K and (b) 1473 K in comparison with experimental data.^75^

Due to the scarce number of experimental datasets for the *α* and *β* phase regions of the Ti-V-O system, a critical quantitative validation of the current work is difficult. In Fig. 5a and b, the calculated isothermal sections at 1073 K and 1473 K are shown in comparison with the measurements by Komjathy.^75^ Qualitatively, the calculations agree with experimental findings that the oxygen solubility increases with increasing temperature. However, the absolute values for the phase boundaries are not in agreement. Fair agreement is seen at 1073 K (Fig. 5a), but the calculated solubility at 1473 K is much larger than the measured solubility (Fig. 5b). As discussed in the previous section, the data from Komjathy are judged to be of low accuracy. Their binary V-O side of the section does not agree with the V-O diagram adopted here,^71^ and the experimental procedure used is not well described. Thus, the accuracy of the data is simply considered too low to motivate the extreme interaction parameter in the ternary system that would be needed to fit to these phase boundaries.

**Fig. 6:**
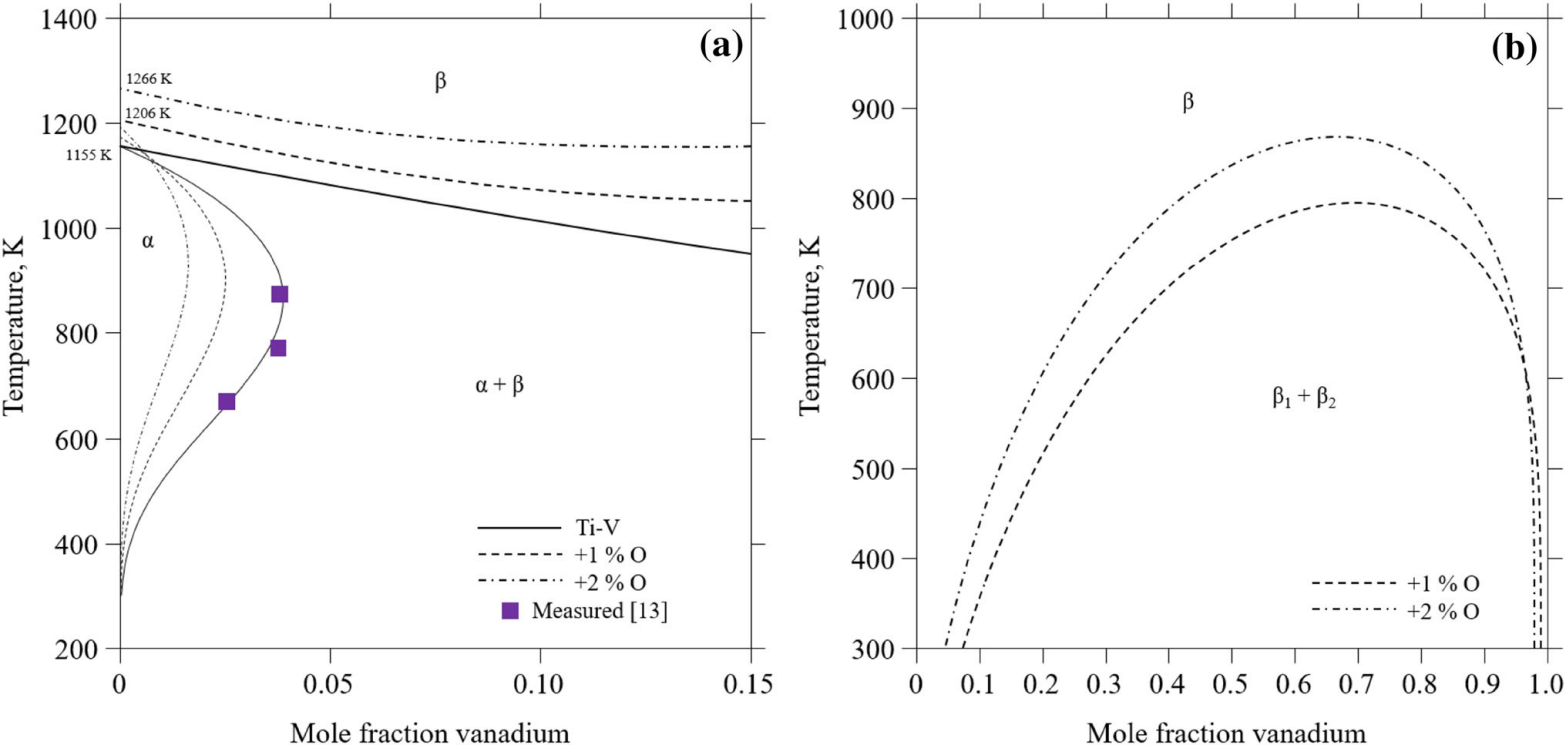
Calculated vertical section for (a) the titanium-rich side of the Ti-V phase diagram with varying oxygen amounts in comparison to experimental data^13^ (symbol), and (b) Metastable diagram at varying oxygen concentrations showing the miscibility gap of the *β* phase

Qualitatively, the calculated behavior of the system at titanium-rich compositions agrees with the literature. In Fig. 6a, the calculated vertical sections along the vanadium content are shown for different Ti-O compositions, i.e., for pure titanium and for titanium with 0.01 mol and 0.02 mol fraction oxygen, respectively. It can be concluded that the vanadium solubility in the *α*-Ti phase decreases with increasing oxygen, as expected from experiments. Furthermore, the *β*-transus temperature is calculated to increase with increasing oxygen content, which again is qualitatively supported by experiments.

Given the different forms of the Ti-V phase diagram published in the past, and the belief that the presence of oxygen could be one of the contributing factors for the inconsistency, it is interesting to look at the metastable phase diagram for the *β* phase when oxygen is added. When no oxygen is added, a metastable miscibility gap in the *β* phase at low temperature, below room temperature, is calculated. The calculated critical temperature is 277 K. Since the model functions are only evaluated from room temperature and above, this should be viewed with some caution. When oxygen is added, the *β* miscibility gap opens up to higher temperatures and to a wider range of vanadium compositions. For example, if 0.01 mol fraction and 0.02 mol fraction oxygen is added, the critical point is calculated to 795 K and 868 K, respectively (Fig. 6b).

The effect of oxygen on the Ti-V system and its influence on the Gibbs energy functions of the phases also changes the calculated *T*_0_ temperatures for the *β* $\to$ *α* transition and the *β* $\to$ *ω* transition. In Fig. 4, the calculated *T*_0_ temperatures for different oxygen additions are shown. The addition of oxygen decreases the *T*_0_ temperature for both transitions. This is in accordance with the work by Paton and Willliams,^42^ in which the *ω*_s_ temperature was shown to decrease for the studied Ti-*x*V (*x* = 0.17, 0.18, 0.19 and 0.20 mass fraction) alloys when the oxygen content was increased. In the case of the lowest oxygen content (about 760 ppm), they determined the *ω*_s_ temperature for the Ti-19 V and Ti-20 V alloys to be much lower than for the Ti-17 V and Ti-18 V alloys. This indicates a very strong composition dependency for *ω*_s_, which ultimately should have been verified by a second set of experiments. Furthermore, the reported experimental details^42^ are sparse and no uncertainties regarding the alloy compositions are given, which would have been helpful when judging the accuracy of the dependency of *ω*_s_ on rather small vanadium variations (0.01 mass fraction). If it is assumed that the low-oxygen-containing alloys in the study by Paton and Williams^42^ are comparable to the binary Ti-V system, the calculated T_0_ line should be above all the experimental data points for the *ω*_s_. This is the case for the Ti-19 V and Ti-20 V alloys but not the Ti-17 V and the Ti-18 V alloys. Also, the measurement of *ω*_s_ by Ikeda et al.,^43^ as well as their estimated *ω*_s_ values, are above the calculated *T*_0_-line. Since determining accurate *ω*_s_ values is a challenging task for the complex metastable phase relationships in the Ti-V system, where even trace amounts of impurities are known to strongly influence phase compositions and diffusivities, these inconsistencies are accepted in the current work.

## Discussion of the Implications of Oxygen on Titanium Alloys for AM

**Fig. 7:**
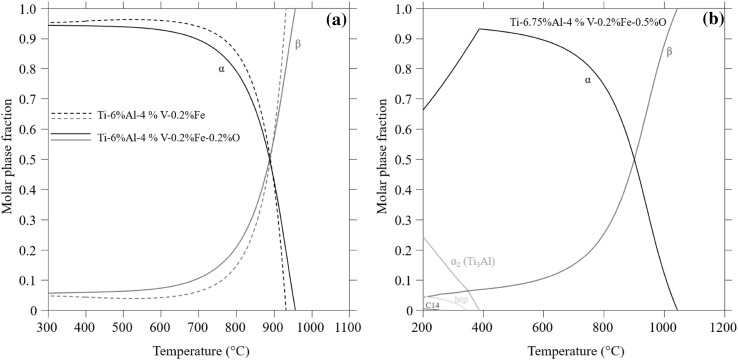
(a) Calculated phase fractions for Ti-6%Al-4%V-0.2%Fe (dashed lines) and Ti-6%Al-4%V-0.2%Fe-0.2%O (solid lines) to show the effect of oxygen on the *α*-*β* phase fraction and *β*-transus temperature. (b) Calculated phase fraction for Ti-6.75%Al-4%V-0.2%Fe-0.5%O to show the stable fraction of *α*_2_ (Ti_3_Al) and a small fraction of a Ti and O-rich hcp phase at lower temperatures

Oxygen influences the mechanical properties of titanium and titanium alloys, e.g. the yield strength of *α*-Ti increases with increasing oxygen. This strength improvement, however, comes at the expense of decreased ductility and toughness and, consequently, depending on application, the tolerance for oxygen content may vary. In the case of AM of titanium alloys, it is thus necessary to keep track of oxygen content in the virgin powder feedstock, account for potential uptake of oxygen during powder handling and printing, and limit the number of powder reuses. It is widely known within the AM community that the oxygen content increases with recycling of titanium powder, e.g. Refs. 78 and 79. This can cause changes in the powder properties, such as flowability, and introduce defects. As shown in the current work, small changes in the oxygen content can also have non-negligible impacts on the microstructure. It is noted that increased oxygen content increases the *β*-transus temperature. In the case of the Ti-6Al-4 V (Ti 6-4) alloy or other near-*α* alloys, this means that *α*-Ti can form at higher temperatures, which may lead to more *α*-Ti in the final microstructure, depending on processing conditions. However, it is also noted that an increase of oxygen affects the equilibrium *β*-phase fraction. In Fig. 7a, the equilibrium phase fractions as a function of temperature are shown for a Ti 6-4 representative composition. The equilibrium *β* fraction increases at temperatures below the *β*-transus when oxygen increases from zero to 0.002 mass fraction, which is the maximum allowed oxygen amount for ASTM Grade 5 Ti 6-4. For the calculations, the Ti-V-O description developed here is combined with ternary descriptions of the Ti-Al-V and Ti-Al-O systems.^80^,^81^ The increase in the equilibrium *β*-phase fraction with increasing oxygen should be accounted for when, e.g. selecting post-treatments for Ti 6-4 alloys manufactured by electron beam melting (EBM) due to long build times at elevated temperatures.

The tolerance for oxygen is also microstructure-dependent. This is discussed by Yan et al.,^82^ where the room-temperature tensile ductility for Ti 6-4 alloys with different oxygen content fabricated by different AM technologies were compared. For similar oxygen levels, AM parts consisting of *α*′ martensite (typical for selected laser melting Ti 6-4) experienced a substantial ductility decrease for oxygen contents above 0.0015 mass fraction, whereas AM parts consisting of a *α *+ *β* microstructure (typical for EBM Ti 6-4) showed good tolerance to oxygen contents up to about 0.004 mass fraction. Hence, AM powder may require different composition specifications depending on the AM technology for which it is produced.

The dramatic decrease in ductility noted for compositions above about 0.004 mass fraction oxygen for a AM Ti 6-4 alloy with a *α *+ *β* microstructure^82^ is interesting to discuss further in respect to the thermodynamics of the system. In Fig. 7b, calculated equilibrium phase fractions as a function of temperature are shown for a ASTM Grade 5 Ti 6-4 composition with an oxygen content of 0.005 mass fraction. At lower temperatures, the ordered *α* phase *α*_2_ (Ti_3_Al) is now an equilibrium phase and, although formation of a phase at these low temperatures is a slow process, it is an indication that ordering of *α* and formation of *α*_2_ is thermodynamically favorable. Such tendencies are thus to be expected at long holding times and slow cooling rates through these temperatures. That oxygen stabilizes *α*_2_ is well known, and a previous study on oxygen-induced microstructural features in as-sintered Ti 6-4^83^ showed that *α*_2_-type nano-sized clusters form in the *α* phase during cooling from the sintering temperature for the sample with 0.0049 mass fraction oxygen. It is well established that *α*_2_ can decrease the ductility in titanium alloys remarkably and, consequently, could be a contributing factor for the ductility loss caused by oxygen in AM Ti 6-4 alloys.

## Conclusion

The thermodynamics of the Ti-V-O system is described using the CALPHAD method. The critical evaluation of available experimental data relevant for the development of the description shows that even small additions of oxygen have a large effect on the thermodynamics of this system. The current work serves as an attempt to take this into account to facilitate quantitative estimations of the effects of oxygen in the Ti-V system. For this purpose, experimental data for the optimization have been carefully selected with special emphasis on the impurity and oxygen levels in titanium-rich systems. Consequently, necessary revisions of the previously published subsystems are performed, i.e., the Ti-O and Ti-V systems.

Equilibrium calculations using the developed description show that small additions of oxygen change phase boundaries notably, while the extension of the *α*-phase field with respect to dissolved V decreases and the *β*-transus temperature is increased, which are all in qualitative agreement with experiments. The calculations also show that the metastable miscibility gap in the *β* phase opens up to much higher temperatures and to a larger alloy composition range when oxygen is added compared to the oxygen-free counterparts. Calculations of *T*_0_ temperatures for both the *β* $\to$ *α* and *β* $\to$ *ω* transitions show a decrease with increasing oxygen addition, which is supported by experiment.

In summary, it can be concluded that the current state of the experimental knowledge of this system and, in particular, the influence of impurities is poor. The large solubility of interstitials, such as oxygen, in titanium makes it safe to conclude that all experimental studies involving titanium will involve the Ti-O system. Furthermore, the most important titanium ore is ilmenite (TiFeO_3_), and, hence, only ultrapure titanium (which is seldom used) can assure that the experiments are not influenced by the presence of iron or other impurities. To further improve the understanding of titanium and titanium alloys, first-principles studies in which truly impurity-free situations as well as controlled impurity amounts can be considered are promising. However, for such calculation approaches to have an impact, they need to be accompanied by experiments on materials of well-defined composition, and, hence, experimental studies of titanium systems with controlled impurity content are encouraged.

Nevertheless, a preliminary version of a thermodynamic description of the Ti–Al-V-O system is applied to discuss the implications of oxygen on the AM processing of titanium alloys. It is suggested that an increase in the *β*-transus temperature due to increased oxygen can be accommodated by tailored post-treatment routes for Ti 6-4, but that oxygen tolerance limits need to be specified based on AM technology and application. In particular, oxygen levels that could cause ordering tendencies and formation of *α*_2_ at service temperatures should be avoided for Ti 6-4.

## References

[1] UK Additive Manufacturing Steering Group, *Additive Manufacturing UK* - *National Strategy 2018*-*25* (2017).

[2] RAMP-UP, *Research Needs and Challenges for Swedish Industrial Use of Additive Manufacturing* (2017).

[3] Anderson IE, White EMH, Dehoff R (2018). Curr. Opin. Solid State Mater. Sci..

[4] Seifi M, Salem A, Beuth J, Harrysson O, Lewandowski JJ (2016). JOM.

[5] Kaufman L, Bernstein H (1970). Computer Calculation of Phase Diagrams with Special Reference to Refractory Metals.

[6] Kattner UR (2016). Tecnol. Em Metal. Mater. e Mineração.

[7] Campbell CE, Kattner UR, Liu Z-K (2014). Integr. Mater. Manuf. Innov..

[8] Ansara I, Dinsdale AT, Rand MH (1998). COST 507 Thermochemical Database For Light Metal Alloys.

[9] Ghosh G (2002). J. Phase Equilibria.

[10] Wang W, Winkler MT, Gunawan O, Gokmen T, Todorov TK, Zhu Y, Mitzi DB (2014). Adv. Energy Mater..

[11] Murray JL (1981). Bull. Alloy Phase Diagrams.

[12] Ermanis F, Farrar PA, Margolin H (1961). Trans. Met. Soc..

[13] Molokanov VV, Chernov D, Budberg P (1977). Met. Sci. Heat Treat..

[14] Murray JL, Smith JF (1989). Phase Diagrams Bin. Vanadium Allloys.

[15] O. Nakano, H. Sasano, T. Suzuki, and H. Kimura, in *Titan.’80 Proceeding 4th Int. Conf. Titanium, Kyoto, Japan* (1980), pp. 2889–2895.

[16] Fuming W, Flower HM (1989). Mater. Sci. Technol..

[17] Chernov D (1975). AY S. Russ. J. Phys. Chem..

[18] Khaled T, Narayanan GH, Copley SM (1978). Metall. Trans. A.

[19] Cuello GJ, Aurelio G, Fernández Guillermet A, Campo J (2002). Appl. Phys. A Mater. Sci. Process..

[20] Adenstedt H, Pequignot J, Raymer J (1952). Trans. Am. Soc. Met..

[21] Ahmed T, Flower HM (1994). Mater. Sci. Technol..

[22] Uesugi T, Miyamae S, Higashi K (2013). Mater. Trans..

[23] Chinnappan R, Panigrahi BK, van de Walle A (2016). Calphad.

[24] Mills KC, Kinoshita K (1973). J. Chem. Thermodyn..

[25] Banerjee S, Mukhopadhyay P (2007). Pergamon Materials Series Vol 12.

[26] Boyer R, Collings EW, Welsch G (1994). Materials Properties Handbook: Titanium Alloys.

[27] McCabe KK, Sass SL (1971). Philos. Mag..

[28] Sikka SK, Vohra YK, Chidambaram R (1982). Prog. Mater Sci..

[29] Hanada S, Izumi A (1986). Metall. Trans. A.

[30] Ming L-C, Manghnani MH, Katahara KW (1981). Acta Metall..

[31] Leibovitch C, Rabinkin A, Talianker M (1981). Metall. Trans. A.

[32] Ghosh C, Basu J, Ramachandran D, Mohandas E (2016). Acta Mater..

[33] Aurelio G, Guiuernw AF, Cuello GJ, Campo J (2002). Metall. Mater. Trans. A.

[34] Silcock JM (1958). Acta Metall..

[35] Aurelio G, Fernández Guillermet A, Cuello GJ, Campo J (2002). Metall. Mater. Trans. A.

[36] Pietrokowsky P, Duwez P (1952). Trans. AIME.

[37] Sato T, Hukai S, Huang YC (1960). J. Aust. Inst. Met..

[38] Kaneko H, Huang YC (1963). J. Japan Inst. Met..

[39] Kaneko H, Huang YC (1963). J. Japan Inst. Met..

[40] Devika M, Ramakrishna Reddy KT, Koteeswara Reddy N, Ramesh K, Ganesan R, Gopal ESR, Gunasekhar KR (2006). J. Appl. Phys..

[41] Mirzayev DA, Ulyanov VG, Shteynberg MM, Protopopov VA (1981). Fiz. Met. Met..

[42] Paton NE, Williams JC (1973). Scr. Metall..

[43] Ikeda M, Komatsu S-Y, Sugimoto T, Kamei K (1990). J. Jpn. Inst. Met..

[44] Zarkevich NA, Johnson DD (2016). Phys. Rev. B.

[45] Mei W, Sun J (2017). MRS Adv..

[46] Yan J-Y, Olson GB (2016). J. Alloys Compd..

[47] Zhang S-Z, Cui H, Li M-M, Yu H, Vitos L, Yang R, Hu Q-M (2016). Mater. Des..

[48] Hampl M, Schmid-Fetzer R (2015). Int. J. Mater. Res..

[49] Cancarevic M, Zinkevich M, Aldinger F (2007). Calphad.

[50] Cao Z, Xie W, Jung IH, Du G, Qiao Z (2015). Metall. Metall. Mater. Trans. B Process Metall. Mater. Process. Sci..

[51] Waldner P, Eriksson G (1999). Calphad.

[52] Murray JL, Wriedt HA (1987). Bull. Alloy Phase Diagrams.

[53] Fischer E (1997). J. Phase Equilibria.

[54] Tetot R, Picard C, Boureau G, Gerdanian P (1978). J. Chem. Phys..

[55] Kubaschewski O, Dench WA (1953). J. Inst. Met..

[56] Jenkins AE, Worner HW (1951). J. Inst. Met..

[57] Jaffee RI, Ogden HR, Maykuth DJ (1950). Trans. Am. Soc. Met..

[58] Bumps ES, Kessler HD, Hansen M (1953). Trans. ASM.

[59] Schofield TH, Bacon AE (1956). J. Inst. Met..

[60] Wasilewski RJ, Kehl GL (1951). J. Inst. Met..

[61] A.D. Mah, K. Kelley, N.L. Gellert, E.G. King, and C.J. O’Brian, *Thermodynamic Properties of Titanium*-*Oxygen Solutions and Compounds*, a technical report with report number BM-RI-5316. (Bureau of Mines, United States, 1955).

[62] Hepworth MT, Schuhmann R (1962). Trans. Met. Soc..

[63] Ariya S, Morozova MP, Volf E (1957). Zhurnal Neogranicheskoi Khimii.

[64] Komarek KL, Silver M (1962). Thermodynamics of Nuclear Materials.

[65] V.A. Reznichenko, F.V. Khalimov, and N.V. Agreev, Protsessy. Proizv. Titana Ego Dvuokisi 193 (1973).

[66] Okabe TH, Suzuki RO, Oishi T, Ono K (1991). Japan Inst. Met..

[67] S. Miyazaki, T. Oishi, K. Ono, G. Lutjering, U. Zwicker, and W. Bunk, in *5th International Conference on Titanium.* (1984), pp. 2657–2663.

[68] N. Sano and F. Tsukihashi, in *69th Committee. JPSP* (1989), p. 31.

[69] Wang W-E, Kim YS (1999). J. Nucl. Mater..

[70] Banus MD, Reed R, Strauss AJ (1972). Phys. Rev. B.

[71] Yang Y, Mao H, Selleby M (2015). Calphad.

[72] Yang Y, Mao H, Chen HL (2017). J. Alloys Compd..

[73] Enomoto M (1996). J. Phase Equilibria.

[74] Lin SS (1967). Titanium-oxygen-vanadium system.

[75] Komjathy S (1961). J. Less-Common Met..

[76] Ageyev NV, Guseva LN, Dolinskaya LK (1975). Russ. Metall..

[77] Li T, Kent D, Sha G, Stephenson LT, Ceguerra AV, Ringer SP, Dargusch MS, Cairney JM (2016). Acta Mater..

[78] Tang HP, Qian M, Liu N, Zhang XZ, Yang GY, Wang J (2015). JOM.

[79] Sun Y, Aindow M, Hebert RJ (2017). Mater. High Temp..

[80] Ilatovskaia M, Savinykh G, Fabrichnaya O (2017). J. Phase Equilibria Diffus..

[81] Zhang F (1997). A Thermodynamic and Experimental Study of the Titanium-Aluminum-Vanadium (Ti-Al-V) Ternary System.

[82] Yan M, Xu W, Dargusch MS, Tang HP, Brandt M, Qian M (2014). Powder Metall..

[83] Yan M, Dargusch MS, Ebel T, Qian M (2014). Acta Mater..

[84] Dinsdale AT (1991). Calphad.

